# Borna disease virus possesses an NF-ĸB inhibitory sequence in the nucleoprotein gene

**DOI:** 10.1038/srep08696

**Published:** 2015-03-03

**Authors:** Akiko Makino, Kan Fujino, Nicholas F. Parrish, Tomoyuki Honda, Keizo Tomonaga

**Affiliations:** 1 Department of Viral Oncology, Kyoto University, Kyoto 606–8507, Japan; 2 Center for Emerging Virus Research, Institute for Virus Research, Kyoto University, Kyoto 606–8507, Japan; 3 Department of Tumor Viruses, Graduate School of Medicine, Kyoto University, Kyoto 606–8507, Japan; 4 Department of Mammalian Regulatory Network, Graduate School of Biostudies, Kyoto University, Kyoto 606–8507, Japan

## Abstract

Borna disease virus (BDV) has a non-segmented, negative-stranded RNA genome and causes persistent infection in many animal species. Previous study has shown that the activation of the IκB kinase (IKK)/NF-κB pathway is reduced by BDV infection even in cells expressing constitutively active mutant IKK. This result suggests that BDV directly interferes with the IKK/NF-κB pathway. To elucidate the mechanism for the inhibition of NF-κB activation by BDV infection, we evaluated the cross-talk between BDV infection and the NF-κB pathway. Using Multiple EM for Motif Elicitation analysis, we found that the nucleoproteins of BDV (BDV-N) and NF-κB1 share a common ankyrin-like motif. When THP1-CD14 cells were pre-treated with the identified peptide, NF-κB activation by Toll-like receptor ligands was suppressed. The 20S proteasome assay showed that BDV-N and BDV-N-derived peptide inhibited the processing of NF-κB1 p105 into p50. Furthermore, immunoprecipitation assays showed that BDV-N interacted with NF-κB1 but not with NF-κB2, which shares no common motif with BDV-N. These results suggest BDV-N inhibits NF-κB1 processing by the 20S proteasome through its ankyrin-like peptide sequence, resulting in the suppression of IKK/NF-κB pathway activation. This inhibitory effect of BDV on the induction of the host innate immunity might provide benefits against persistent BDV infection.

Borna disease virus belongs to the order *Mononegavirales* and possesses a non-segmented, negative-stranded RNA genome. The characteristic properties of this virus include its broad host range in vertebrates^1^,^2^,^3^ and its ability to establish persistent infection in the cell nucleus^4^,^5^,^6^. The BDV genome encodes 6 genes in the following 3′ to 5′ order: nucleoprotein (N), phosphoprotein (P), X, matrix protein (M), glycoprotein (G), and polymerase protein (L)^7^,^8^,^9^. The M protein undercoats the viral envelope^10^,^11^, and the G protein mediates viral entry into host cells^12^,^13^,^14^. The N, P, and L proteins form the polymerase complex and perform roles in the transcription and replication of the viral genome^15^,^16^,^17^.

Most likely due to the 3′ to 5′ transcription initiation gradient^8^, the N protein is the most abundant viral proteins in acutely infected cells^18^ and animals^19^; it is also the dominant target of the humoral^20^ and CD8^+^-T-cell-mediated immune reactions^21^. In most infected cells, the N protein is concentrated in viral replication factories in the nucleus^22^; however, it is capable of nucleocytoplasmic shuttling due to a nuclear export signal as well as a nuclear localization signal^23^. These properties make N a strong candidate for a viral gene that influences host cell functions, including innate immunity.

When a cell is infected with a virus, pattern recognition receptors (PRRs) rapidly sense non-self nucleic acids and proteins, leading to the activation of the antiviral innate immune response^24^. One key downstream component of this response, type I interferon, has been shown to inhibit BDV infection in a number of experimental systems^25^,^26^. Given the wide range of cells in which BDV effectively establishes persistent infection, it is unsurprising that it has evolved many strategies to avoid triggering PRRs as well as to interrupt their signaling cascades. BDV modifies the termini of its RNA genome in a way that avoids recognition by the cytoplasmic innate receptor RIG-I^27^,^28^. In addition, BDV can inhibit MAVS, a molecule important for activating transcription factors, including IRF3 and 7, after PRR engagement^29^. Finally, BDV inhibits TBK-1, a kinase needed to phosphorylate IRF3 and 7, which allows them to enter the nucleus to transcribe interferon and other innate immune effectors^30^. Thus, BDV avoids or counteracts the innate immune response at multiple levels. Innate immune pathways are often redundant and partially overlapping; thus, BDV may utilize additional unknown mechanisms to thoroughly antagonize the host response. For example, BDV is inhibited by the constitutive activation of NF-κB^31^, but no BDV proteins that interact with this molecule have been identified.

NF-κB is a transcription factor involved in immune induction, embryonic development, and cell proliferation in response to various extracellular stimuli^32^,^33^. The NF-κB family consists of five genes, NF-κB1, NF-κB2, RelA, RelB, and c-Rel, which form homo- or hetero-dimers in the cytoplasm^34^,^35^. When the NF-κB signaling pathway is activated, IκB, which is an inhibitory factor of NF-κB, is phosphorylated by IκB kinase (IKK) and degraded by the 20S proteasome^36^. This process frees NF-κB to translocate into the nucleus, where it behaves as a transcription factor^32^,^33^. When cells expressing constitutively active IKK are stimulated with tetradecanoyl phorbol acetate, a potent NF-κB signaling pathway stimulus, NF-κB activation is lower in BDV-infected cells than in mock cells. This difference suggests that BDV infection suppresses the IKK/NF-κB signaling pathway downstream of IKK^31^.

To better understand how BDV evades innate immunity and establishes persistent infection, we first confirmed that BDV suppresses NF-κB activation, then aimed to determine the mechanism by which it does so. It has previously been reported that certain cell-penetrating peptides with sequence motifs in common with specific NF-κB family genes can competitively inhibit the NF-κB signaling pathway^37^,^38^,^39^. Furthermore, a viral peptide derived from the vaccinia virus A46 protein (VIPER) inhibits the Toll-like receptor 4 (TLR4) signaling pathway, which also mediates signal transduction using NF-κB by binding to the BB loop of Toll/interleukin-1 receptor (TIR) domains of the receptor and adaptor, thus disrupting the receptor-adaptor interaction^40^,^41^. We thus searched the BDV proteins for motifs shared with the NF-κB family proteins, aiming to identify NF-κB inhibitory peptide sequences to elucidate the mechanism by which BDV suppresses NF-κB activation.

Here, we show that BDV-N shares a common ankyrin-like motif with NF-κB1. In cells, this BDV-N-derived peptide inhibits the NF-κB signaling pathway. The inhibitory peptide derived from BDV-N demonstrates another mechanism used by BDV to overcome innate immunity and establish persistent infection.

## Results

### BDV infection inhibits NF-κB activation

**Figure 1:**
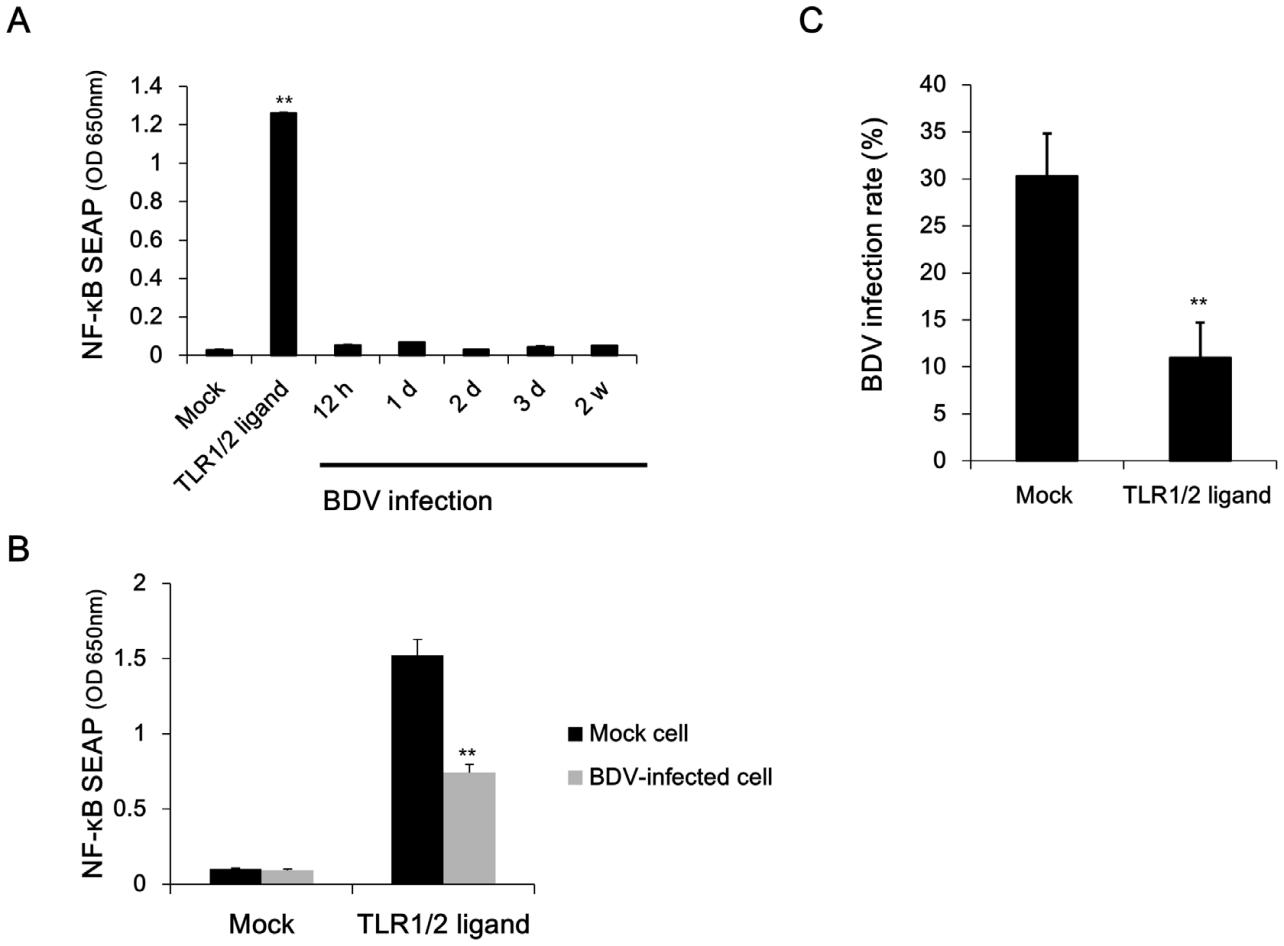
BDV infection suppresses NF-κB activation. (A) THP1-CD14 cells were infected with cell-free rBDV P/M-GFP at a multiplicity of infection of 0.1, and the resulting supernatants were collected. The SEAP activity was determined at the indicated time points (N = 3). (B) THP1-CD14 cells infected with rBDV P/M-GFP were stimulated with 100 ng/ml of TLR1/2 ligand (Pam3CSK4). At 48 h post-stimulation, GFP-positive cells were measured using an image-based cytometer. Error bars represent standard deviation of the mean (N = 3). **: *P* = 0.00474 (one-tailed t-test) (C) THP1-CD14 cells infected with rBDV P/M-GFP were stimulated with 100 ng/ml of TLR1/2 ligand (Pam3CSK4). The SEAP activities of the supernatants were determined at 24 h post-stimulation. Error bars represent the standard deviation of the mean (N = 3). **: *P* = 0.00761 (one-tailed t-test)

To first assess the reproducibility of the previous data that BDV suppresses the processing and activation of NF-κB during infection^31^, we employed THP1-CD14 cells^42^. These cells express secreted alkaline phosphatase (SEAP) under the control of a promoter that is activated by NF-κB. THP1-CD14 cells were infected with rBDV P/M-GFP^43^, and the resulting SEAP activity was measured at from 12 h to 2 weeks post-infection. The SEAP activity in the culture supernatant was not significantly elevated above the level from mock-infected cells at any point over the 2 weeks (Figure 1A), suggesting a lack of detectable NF-κB activation induced by BDV infection in THP1-CD14 cells. In contrast, the addition of the positive control, the TLR1/2 agonist Pam3CSK4, at a 100 ng/ml concentration for 24 hours led to readily detectable SEAP production. These data could indicate that BDV avoids recognition by PRRs that signal through NF-κB or, alternatively, that BDV may be recognized by such PRRs but their signaling is interrupted prior to the activation of NF-κB. To distinguish between these alternatives, we attempted to activate NF-κB in persistently BDV-infected cells using Pam3CSK4. BDV-infected cells secreted less SEAP than mock cells when activated with Pam3CSK4 (Figure 1B), suggesting that BDV-infected cells actively suppress NF-κB signaling. To assess whether NF-κB activation has a deleterious effect on viral replication, we activated NF-κB using Pam3CSK4 in cultures of THP1-CD14 cells that had been exposed to BDV 7 days earlier. Forty-eight hours after stimulation, we measured the extent of infection by determining the percentage of GFP-expressing cells. NF-κB activation with Pam3CSK4 resulted in significantly fewer BDV-infected cells in the culture (Figure 1C). These data are consistent with a previous report^31^ and indicate that BDV infection inhibits NF-κB activation, which could otherwise impair BDV replication.

### Inhibition of NF-κB activation by a peptide derived from BDV-N

**Figure 2:**
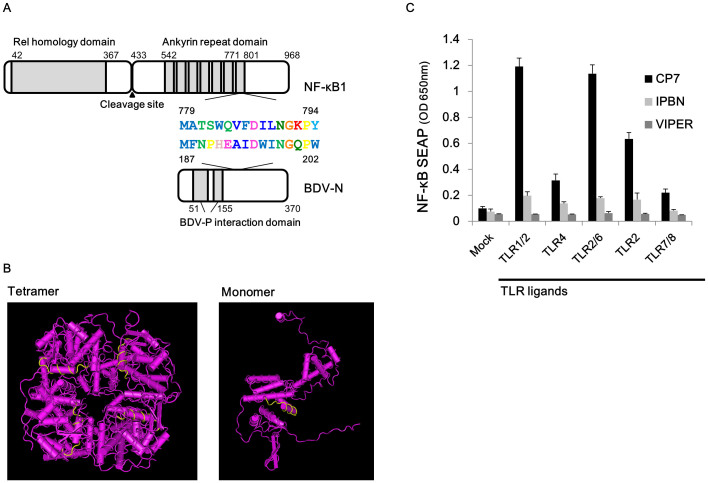
Inhibitory effect of the virus peptide on NF-κB activation. (A) Schematic diagram of NF-κB1 and BDV-N. (B) The crystallographic structure of the BDV-N tetramer and monomer (Protein Databank # 1N93). Yellow lines indicate the IPBN. (C) Inhibitory effect of the viral peptide on NF-κB activation. THP1-CD14 cells were pre-treated with 100 μg/ml of the viral peptide derived from BDV-N, a negative control peptide (CP7), or the positive control peptide (VIPER) and stimulated with five TLR ligands. At 24 h post-stimulation, the SEAP activities in the supernatants were measured. Error bars represent the standard deviation of the mean (N = 3).

Based on previous reports that peptides possessing common sequence motifs with specific NF-κB family genes competitively inhibit NF-κB signaling^37^,^38^,^39^ and that VIPER derived from vaccinia virus A46 protein inhibits TLR4 signaling^40^,^41^, we screened for common motifs between BDV genes and the human NF-κB family to identify possible mechanisms underlying the observed inhibition of NF-κB activation in BDV-infected cells. For this purpose, we used Multiple EM for Motif Elicitation (MEME) analysis, a tool for the discovery of common motifs across amino acids sequences that uses the MM algorithm^44^,^45^. We detected several shared motifs (Table 1). We focused on a motif shared between BDV-N and NF-κB1 because both the length of the motif sequence (16 amino acids) and the localization of the motif (ankyrin repeat domain of NF-κB1^32^,^33^; exposed domain of BDV-N tetramer and monomer^46^) suggested that it could have a biologically relevant effect (Figure 2A and B).

To assess the inhibitory effect of the identified motif in BDV-N on NF-κB activation, THP1-CD14 cells were incubated with 100 μg/ml of the viral peptide fused with a cell-penetrating peptide (nine arginine residues) at the C-terminus for 4 h. We then stimulated the cells with five different TLR ligands known to activate NF-κB, predicting that a direct interaction of the BDV-N peptide with NF-κB would block SEAP production regardless of the upstream pathway involved. Indeed, 24 h after stimulation with TLR ligands, NF-κB activation by all ligands was significantly suppressed in cells pre-treated with virus peptides derived from BDV-N (*P* = 0.00168 for TLR1/2; *P* = 0.01198 for TLR4; *P* = 0.00090 for TLR2/6; *P* = 0.00862 for TLR2; *P* = 0.01337 for TLR7/8), as well as with the positive control VIPER (*P* = 0.00084 for TLR1/2; *P* = 0.00823 for TLR4; *P* = 0.00131 for TLR2/6; *P* = 0.00203 for TLR2; *P* = 0.00646 for TLR7/8) (Figure 2C). In contrast, NF-κB signaling was highly activated by all TLR agonists in cells pre-treated with the control peptide CP7. These data suggest that the identified viral peptide inhibits NF-κB activation. We therefore refer to this peptide as the Inhibitory Peptide derived from BDV-N (IPBN).

### BDV-N and the peptide derived from BDV-N reduce NF-κB1 processing by the 20S proteasome

**Figure 3:**
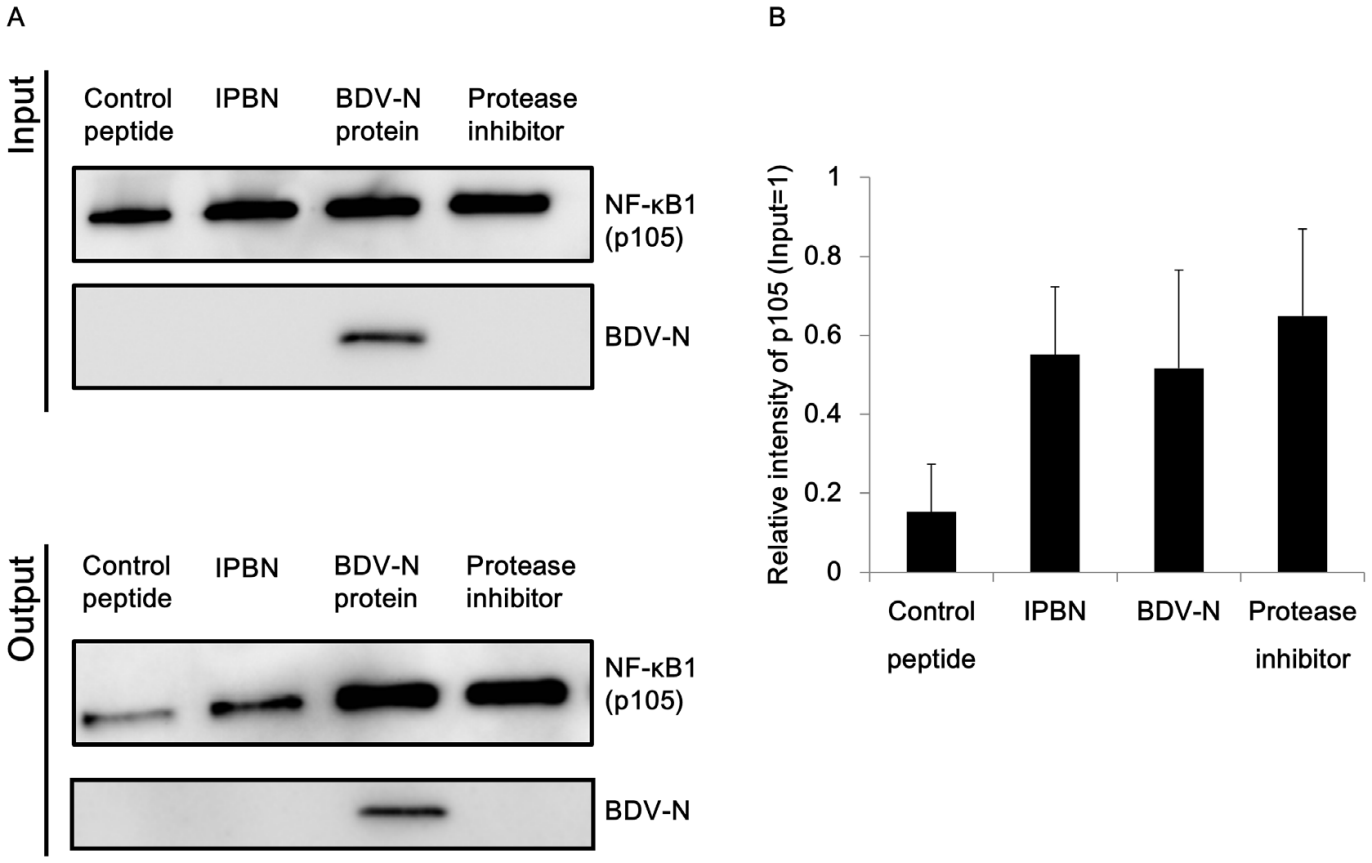
The 20S proteasome assay. (A) NF-κB1 protein and BDV-N protein, the virus peptide derived from BDV-N or 1 X complete protease inhibitor cocktail, were incubated with 20S proteasome at 37°C for 1 h. To detect the p105 form of NF-κB1, SDS-PAGE and western blotting with anti-FLAG M2 monoclonal antibody were performed. BDV-N was detected using an anti-HA monoclonal antibody. Full-length blots are presented in Supplementary Figure 2. (B) The intensities of the p105 bands were quantified by ImageJ. The relative amount of the output p105 compared with the input p105 was calculated. The error bars represent the standard deviation of the mean (N = 3).

The ankyrin repeat domain of NF-κB1 represses its transcription activity. Upon NF-κB pathway activation, NF-κB1 is phosphorylated, which promotes the processing of the ankyrin repeat-containing C-terminal region by the 20S proteasome and produces a shift in the molecular weight from 105 to 50^36^. We hypothesized that IPBN, and perhaps also intact BDV-N, could inhibit NF-κB signaling by preventing this processing. To determine whether IPBN affects the processing of NF-κB1 p105 into p50, we performed an *in vitro* 20S proteasome assay using IPBN and affinity-purified recombinant BDV-N and NF-κB1 proteins, as previously reported^36^. As shown in Figure 3, when NF-κB1 was incubated with the control peptide and then with the 20S proteasome, p105 was markedly reduced, confirming NF-κB1 processing by the 20S proteasome. This processing could be effectively blocked using a protease inhibitor cocktail. Recombinant BDV-N and IPBN suppressed the processing of p105 as efficiently as the protease inhibitor (*P* = 0.03401 for BDV-N; *P* = 0.03904 for IPBN; *P* = 0.04556 for protease inhibitor) (Figure 3A and B). These data suggest that BDV-N and IPBN inhibit NF-κB activation by preventing NF-κB1 processing by the 20S proteasome, allowing unprocessed NF-κB1 to act as IκB^37^,^38^, the inhibitory factor of NF-κB.

### The IPBN sequence is dispensable for the interaction of BDV-N with NF-κB1

**Figure 4:**
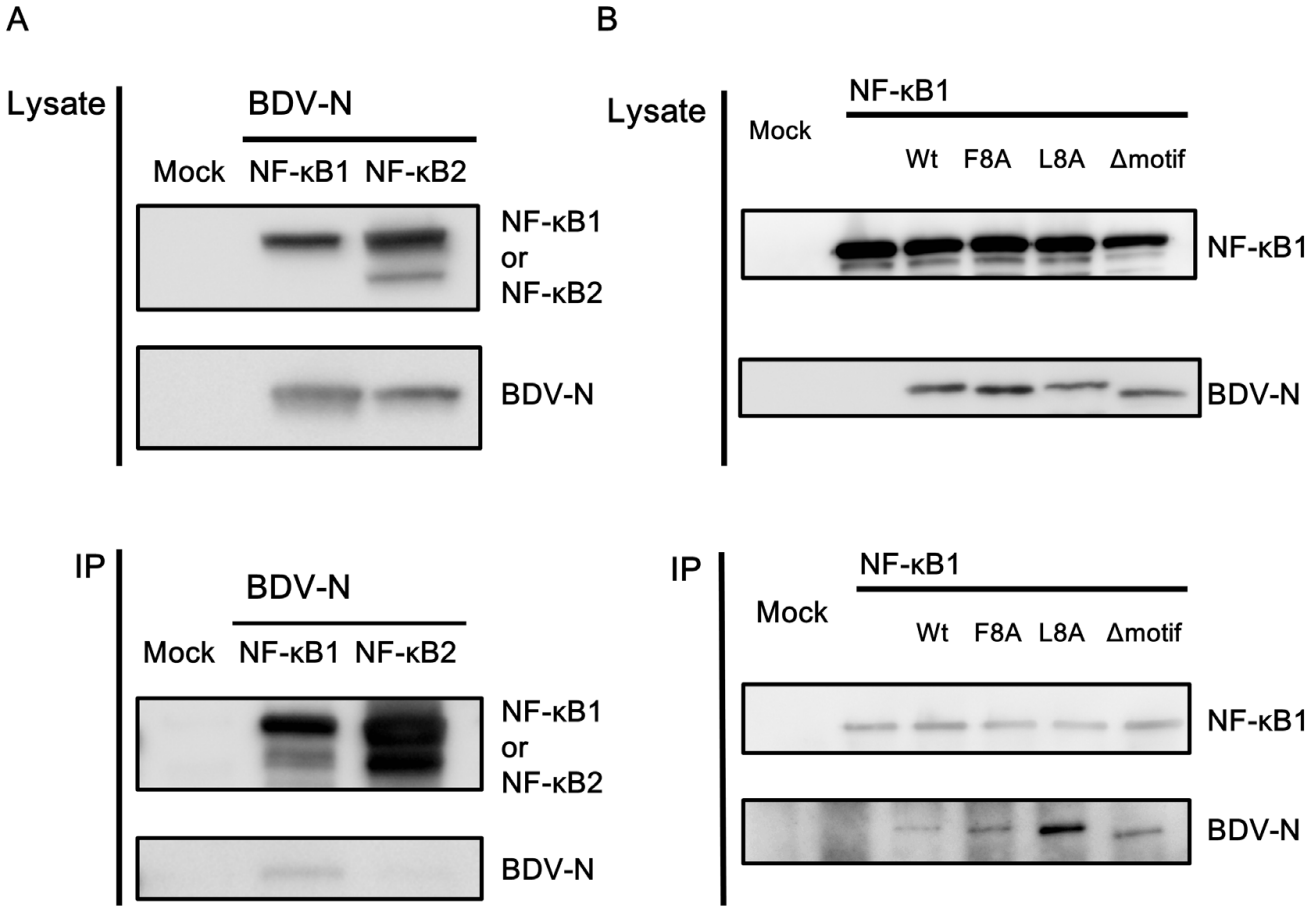
Interaction of BDV-N with NF-κB1. (A) Lysates from cells expressing HA-tagged BDV-N were mixed with lysates from cells transfected with FLAG-tagged NF-κB1 or FLAG-tagged NF-κB2 for 30 min at 4°C. Mixed cell lysates were then immunoprecipitated with an anti-FLAG M2 monoclonal antibody using Protein G Dynabeads. SDS-PAGE and western blotting with an anti-HA monoclonal antibody and anti-FLAG M2 monoclonal antibody were performed. Full-length blots are presented in Supplementary Figure 3. (B) Lysates from cells expressing HA-tagged BDV-N or HA-tagged IPBN-alanine substitution in the first 8 (F8A) or last 8 (L8A) amino acids or -deletion mutants (Δmotif) of BDV-N were mixed with lysates from cells and transfected with the FLAG-tagged NF-κB1 for 30 min at 4°C. The mixed cell lysates were then subjected to an IP assay with anti-FLAG M2 monoclonal antibody using Protein G Dynabeads. Full-length blots are presented in Supplementary Figure 4.

We focused our investigation on the BDV-N gene, as it contains a peptide with similarity to the ankyrin repeat domain of NF-κB. This peptide alone is capable of inhibiting NF-κB signaling; however, intact BDV-N protein also inhibits the processing of NF-κB1. Therefore, we determined whether intact BDV-N interacts with NF-κB1 using an immunoprecipitation assay. Lysates from cells expressing HA-tagged BDV-N and FLAG-tagged NF-κB1 or FLAG-tagged NF-κB2 were mixed for 30 min at 4°C, and the mixed cell lysates were immunoprecipitated with an anti-FLAG antibody (SIGMA). As shown in Figure 4A, BDV-N was co-immunoprecipitated with NF-κB1, but not with NF-κB2, which shares no common motif with BDV-N. This result indicates that BDV-N interacts specifically with NF-κB1. Interestingly, we found that alanine substitution or deletion of the IPBN amino acid sequence in BDV-N had no effect on co-immunoprecipitation with NF-κB1 (Figure 4B). Note that the increased binding property of L8A mutant to NF-κB1 might be resulted from its loose tetramer formation by the alanine replacement. In fact, the L8A mutant has four amino acid changes in the residues involved in the oligomerization of BDV-N, while F8A contains only one change^46^. Thus, the identified motif sequence is dispensable for the interaction of BDV-N with NF-κB1, suggesting that BDV-N interacts with NF-κB1 via additional domains not apparent at the level of motif similarity.

## Discussion

The ankyrin repeat domain of NF-κB1 binds to its Rel homology region, which leads to the repression of transcription factor activity (Figure 2A). The phosphorylation of NF-κB1 (p105) by signal transduction pathways induces the processing of its ankyrin repeat domain by the 20S and 26S proteasomes, and the mature p50 form gains transcription factor activity^36^,^47^. This study revealed that BDV has an ankyrin-like motif in common with NF-κB1 in its N gene. This peptide and intact BDV-N inhibit the processing of NF-κB1 from p105 to p50 by the 20S proteasome, suppressing NF-κB activation. At present, it is unclear exactly how BDV-N suppresses NF-κB1 processing by the 20S proteasome. However, we observed a direct interaction between BDV-N and NF-κB1. Furthermore, BDV-N itself was not degraded by the 20S proteasome, suggesting that it does not act as a decoy for NF-κB1 processing. The IPBN sequence was not necessarily required for the interaction between BDV-N and NF-κB1 (Figure 4B), indicating that at least one other domain within N is necessary to mediate this interaction. Together, our data suggest that the close proximity of BDV-N to NF-κB1 might be important for the efficient inhibitory effects of BDV-N on NF-κB activation. Our working hypothesis is that docking BDV-N onto NF-κB1 might mimic a longer ankyrin repeat domain, which has been reported to exert a suppressive effect on NF-κB1 processing^47^. Structural analysis shows that the ankyrin-like motif is exposed in the BDV-N tetramer and monomer^46^, consistent with this hypothesis (Figure 2B). However, our report has several limitations. At first, we couldn't detect the mature p50 from in the 20S proteasome assay, because of the failure to purify the recombinant NF-κB1 protein tagged at N-terminus, in addition to the low reactivity of the anti-p50 antibodies. We were also unable to test whether BDV-N interacts with NF-κB in infected cells and to rescue recombinant BDV, which has no IPBN. Furthermore, because the interaction of BDV-N with NF-κB1 is not strictly dependent on the identified peptide, we are still exploring strategies to disrupt this interaction while maintaining other critical virological functions of N, which would allow us to definitively assess the relevance of this interaction to BDV infection and persistence. We are now trying to rescue serial mutants of BDV via alanine substitution in IPBN. Further investigation is needed to elucidate the detailed mechanism of the inhibitory effect of BDV-N and IPBN on NF-κB1.

We successfully identified the shared motif between BDV-N and NF-κB1 using MEME analysis, which is a web server for discovering common motif(s) in DNA or protein sequences through the MM algorithm, an extension of the expectation maximization technique^44^,^45^. The identified motif was found to be well-conserved in both the BDV and avian bornavirus nucleoproteins. In 202 of the sequences available in GenBank at the time of submission, only two conservative changes (M187V and G199A) were present at higher than 15%. Indeed, the peptide generated from the identified motif efficiently suppressed NF-κB activation, indicating that MEME analysis is a useful tool in the search for antagonized motifs of viral protein to host genes. In addition to IPBN, we also found that the V protein of the Measles virus shares a motif with the TIR domains of the TIR-domain-containing adapter-inducing interferon-β (TRIF)-related adaptor molecule (TRAM) and myeloid differentiation primary-response gene 88 (MyD88)-adaptor-like (MAL), which are the adaptor proteins in the TLR signaling pathway^48^. The identified virus peptide (DRWCNPMC), which fused with the cell-penetrating peptide at the C-terminus, showed inhibitory effects on some of the TLR signaling pathways (Figure S1; *P* = 0.00105 for TLR1/2; *P* = 0.10236 for TLR4; *P* = 0.00475 for TLR2/6; *P* = 0.00111 for TLR2; *P* = 0.00596 for TLR7/8). These data also strongly support the usefulness of this strategy.

Another interesting finding of this study is that the VIPER peptide that we chose as a positive control inhibited NF-κB signaling through all TLR agonists examined, as it has previously been reported to be specific for the TLR4 pathway^40^,^41^. However, we believe that our results are valid and suggest a previously unrecognized breadth of VIPER inhibition. As shown in Figure 2, a control peptide did not inhibit signaling by any of the agonists tested. The identified peptide from the V protein of the Measles virus did not inhibit NF-κB signaling by the TLR4 agonist and had approximately half the inhibitory effect of either IPBN or VIPER on the other pathways (Figure S1). Together with the control peptide data, this result argues against a systemic bias toward pan-inhibition in our experimental system.

BDV antagonizes host innate immune functions in many ways: the genomic RNA of BDV escapes RIG-I recognition through the processing of the 5′ termini^27^,^49^; BDV-P counteracts Traf family member associated NF-κB activator-binding kinase 1 (TBK-1)-dependent IFN-β expression by acting as a competitive inhibitor of TBK-1^30^; and BDV-X inhibits mitochondrial antiviral signaling protein-induced apoptosis^29^. By showing for the first time that BDV interacts with and inhibits NF-κB, a transcription factor used by many innate immune pathways to transduce signals into the nucleus, this study provides a key insight into how BDV might establish its characteristic persistent infection in the cell nucleus.

In summary, we have identified a viral peptide that shares motifs with and inhibits NF-κB, validating the use of the MEME tool for this purpose. This peptide and other peptides identified in this way could prove useful as pharmacological agents. IPBN inhibits NF-κB, which is being explored as a therapy for many diseases, such as cancer, asthma, and muscular dystrophy^32^,^50^. Furthermore, this result provides a starting point for dissecting virus/host protein interactions. Future studies of BDV-N/NF-κB interaction are warranted to better understand the strategies used by bornaviruses, which have been infecting vertebrate hosts for millions of years^51^, to counter innate immunity.

## Methods

### Cells, virus, and peptides

THP1-CD14 cells (InvivoGen, San Diego, CA, USA) were maintained in Roswell Park Memorial Institute 1640 medium (SIGMA-ALDRICH Japan, Tokyo, Japan) with 10% fetal calf serum (FCS), 1X Penicillin-Streptomycin Solution (Wako, Osaka, Japan), 200 μg/ml of Zeocin™ (Life Technologies Japan, Tokyo, Japan), 250 μg/ml of G418 (InvivoGen), and 100 μg/ml of Normocin (InvivoGen). The 293T cells and BDV-infected Vero cells were grown in Dulbecco's modified Eagle's minimal essential medium (Life Technologies Japan) with 10% FCS and 1X Penicillin-Streptomycin Solution.

The virus used in this study was recombinant BDV, which expresses GFP inserted between the P and M genes of BDV strain He/80, named rBDV P/M-GFP^43^,^52^. To prepare cell-free rBDV P/M-GFP, Vero cells persistently infected with rBDV P/M-GFP were sonicated (BioRuptor UCD-300) and centrifuged at 1200 × *g* for 25 min at 4°C. The supernatant was collected and used as cell-free rBDV P/M-GFP.

The virus-derived peptide (MFNPHEAIDWINGQPW), control peptide (RNTISGNIYSA), and VIPER (KYSFKLILAEY) with cell-penetrating peptide (nine arginine residues) at the C-terminus were custom synthesized (Life Technologies Japan).

### MEME analysis

Amino acid sequences of all BDV genes were submitted to the MEME web server (http://meme.nbcr.net/meme/cgi-bin/meme.cgi) along with one gene from each of the five human NF-κB families. The motif search criteria were set as follows: the width of the motif ranged from 6 to 50 AA, with a maximum of 3 motifs per gene and a maximum of 2 sites per motif.

### NF-κB activation assay

THP1-CD14 cells express TLRs and CD14 at high levels and express the SEAP gene in a NF-κB-dependent manner. To assess the SEAP production, the culture supernatant of THP1-CD14 cells was incubated with QUANTI-Blue™ (InvivoGen) for 1 h at 37°C, after which the optical density (OD) at 650 nm was measured using a Microplate reader SH-9000 (CORONA ELECTRIC Co., Ltd). To assess the NF-κB activation by BDV infection, THP1-CD14 cells were infected with cell-free rBDV P/M-GFP at a multiplicity of infection of 0.1, and the supernatant was harvested and SEAP measured at 12 h, 1 d, 2 d, 3 d, and 2 weeks post-infection. To evaluate the effect of NF-κB activation on BDV infection, a culture of THP1-CD14 cells approximately 30% infected with rBDV P/M-GFP was stimulated with 100 ng/ml of the TLR1/2 ligand. GFP-positive cells were measured using a Tali® Image Cytometer (Life Technologies Japan) at 48 h post-stimulation, and the SEAP activity was assessed at 24 h post-stimulation.

To evaluate the inhibitory effects of the virus peptide on NF-κB activation, 2 × 10^5^ THP1-CD14 cells were incubated with 100 μg/ml of each peptide for 4 h at 37°C, then subjected to stimulation with 100 ng/ml of Pam3CSK4, 10 ng/ml of TLR4 ligand (LPS), 10 ng/ml of TLR 2/6 ligand (FSL-1), 10^7^ cells/ml of TLR2 ligand (heat killed *L. Monocytogenes*), and 5 μg/ml of TLR 7/8 ligand (CL075) (all from InvivoGen), respectively. At 24 h after stimulation, the SEAP activity of the supernatant was determined as described above. The results shown represent the means of three independent experiments. The P values were calculated using a one-tailed t-test.

### The 20S proteasome assay

We performed the 20S proteasome assay as previously described^36^. Briefly, BDV-N and NF-κB1 proteins were purified with antibodies using Dynabeads Protein G (Life Technologies Japan). The NF-κB1 protein and BDV-N protein, IPBN, or 1 X complete protease inhibitor cocktail (Roche Diagnostic K.K., Tokyo, Japan) were incubated with the 20S proteasome (Boston Biochem, Cambridge, MA, USA) at a molecular ratio of 25:25:1 in a buffer solution (20 mM Tris pH 7.0, 250 mM NaCl, 10 mM MgCl_2_, and 1 mM DTT) at 37°C for 1 h. The resultant proteins were subjected to SDS-PAGE and western blotting with an anti-FLAG M2 monoclonal antibody (SIGMA-ALDRICH Japan) to detect the p105 form of NF-κB1. The p50 form was not detected because of the cleavage of the C-terminal FLAG during 20S processing. BDV-N was detected using an anti-HA monoclonal antibody.

### Immunoprecipitation (IP) assay

For the IP assay, 293T cells were transfected with pcDNA3 encoding BDV-N, alanine substitution mutants in the first 8 (F8A) or last 8 (L8A) amino acids of IPBN, or IPBN deletion mutants of BDV-N tagged with HA at N-terminus, pCMV6 expressing NF-κB1 fused with FLAG-tag at C-terminus (OriGene Technologies, Inc., Rockville, MD, USA), and pCMV6 expressing NF-κB2 fused with FLAG-tag at C-terminus (OriGene Technologies, Inc.), and the cells were then lysed in a buffer containing 20 mM Tris-HCl pH 7.4, 150 mM NaCl, 1% Triton-X, 1 mM EDTA, and 1 X complete protease inhibitor cocktail. Cell lysate expressing HA-tagged BDV-N or BDV-N mutants was mixed with cell lysate transfected with FLAG-tagged NF-κB1 or FLAG-tagged NF-κB2 for 30 min at 4°C, and the mixed cell lysates were immunoprecipitated with anti-FLAG M2 monoclonal antibody using Protein G Dynabeads, according to the manufacturer's instructions. The mixed cell lysates and IP products were subjected to SDS-PAGE and western blotting using an anti-HA monoclonal antibody (Abcam, Cambridge, UK) and an anti-FLAG M2 monoclonal antibody.

## Supplementary Material

Supplementary Information Supplementary information

## Figures and Tables

**Table 1:** Extracted motifs

NF-κB Family	BDV gene	Motif sequence
NF-κB1	N	BDV-N ^187^MFNPHEAIDWINGQPW^202^
		NF-κB1 ^779^MATSWQVFDILNGKPY^794^
NF-κB2	P	BDV-P ^145^MKTMMETM^162^
		NF-κB2 ^143^KKNMMGTM^160^
RelA	Not extracted	
RelB	N	BDV-N ^186^QMFNPH^191^
		RelB ^556^NMFPNH^561^
c-Rel	G	BDV-G ^115^DPFECNWFYCC^125^
		c-Rel ^560^DAFEGSDFSCA^571^
