# Supplementary material for: Borna disease virus possesses an NF-ĸB inhibitory sequence in the nucleoprotein gene
Source: Sci Rep. 2015 Mar 3;5:8696. doi: 10.1038/srep08696 (PMC4649702; doi:10.1038/srep08696)
Supplement: Supplementary Information [file srep08696-s1.pdf]

Supplementary Information

Title Borna disease virus possesses an NF-κB inhibitory sequence in the nucleoprotein gene.

Author names Akiko Makino[1,2†], Kan Fujino[1*], Nicholas F. Parrish[1], Tomoyuki Honda[1,3], Keizo Tomonaga[1,3,4†]

Affiliations

[1]Department of Viral Oncology, and [2]Center for Emerging Virus Research, Institute for Virus Research, [3]Department of Tumor Viruses, Graduate School of Medicine, [4]Department of Mammalian Regulatory Network, Graduate School of Biostudies, Kyoto University, Kyoto 606-8507, Japan.

†Authors for correspondence

Email; tomonaga@virus.kyoto-u.ac.jp, akkmakino@gmail.com

[*]Present address; Department of Microbiology II, School of Veterinary Medicine, Azabu University, Fuchinobe, Chuo-ku, Sagamihara-shi, Kanagawa, 252-5201, Japan

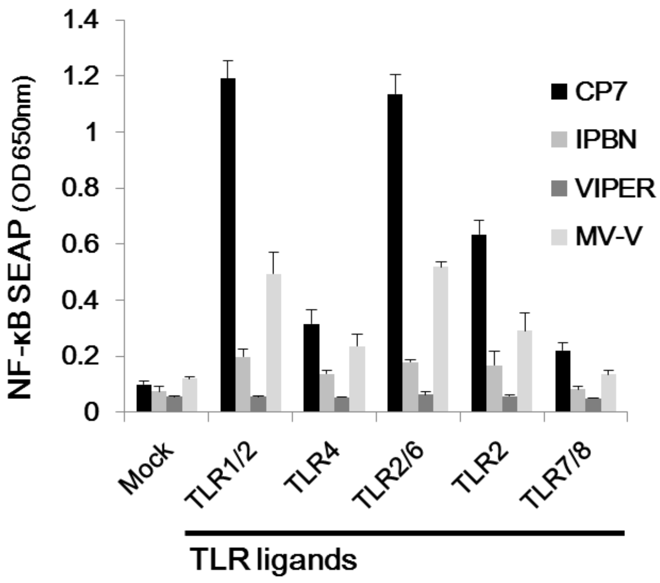

Figure S1 Inhibitory effect of peptide derived from Measles virus V protein on NF-κB activation.

THP1-CD14 cells were pre-treated with 100 μg/ml of IPBN, the peptide derived from Measles virus V protein (MV-V), the negative control peptide (CP7), or the positive control peptide (VIPER), and stimulated with five kinds of TLR ligands, respectively. At 24 h post-stimulation, SEAP activities in the supernatants were measured. Error bars represent standard deviation of the mean (N=3). The result of IPBN, CP7, and VIPER pre-treatments are same as shown in Figure 2C.

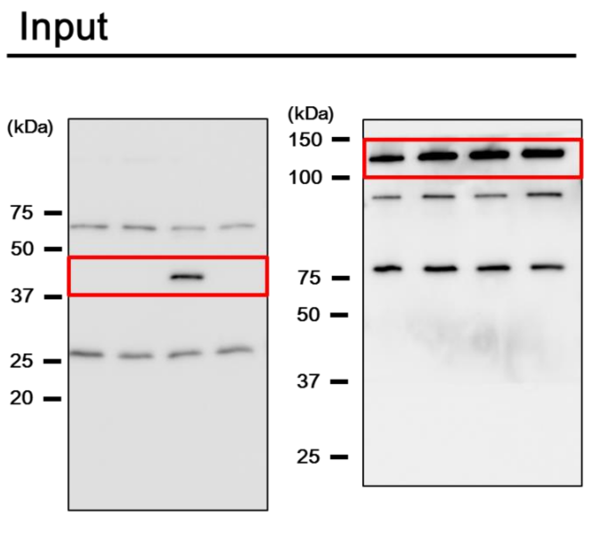

Figure S2 Full-length blots of Figure 3

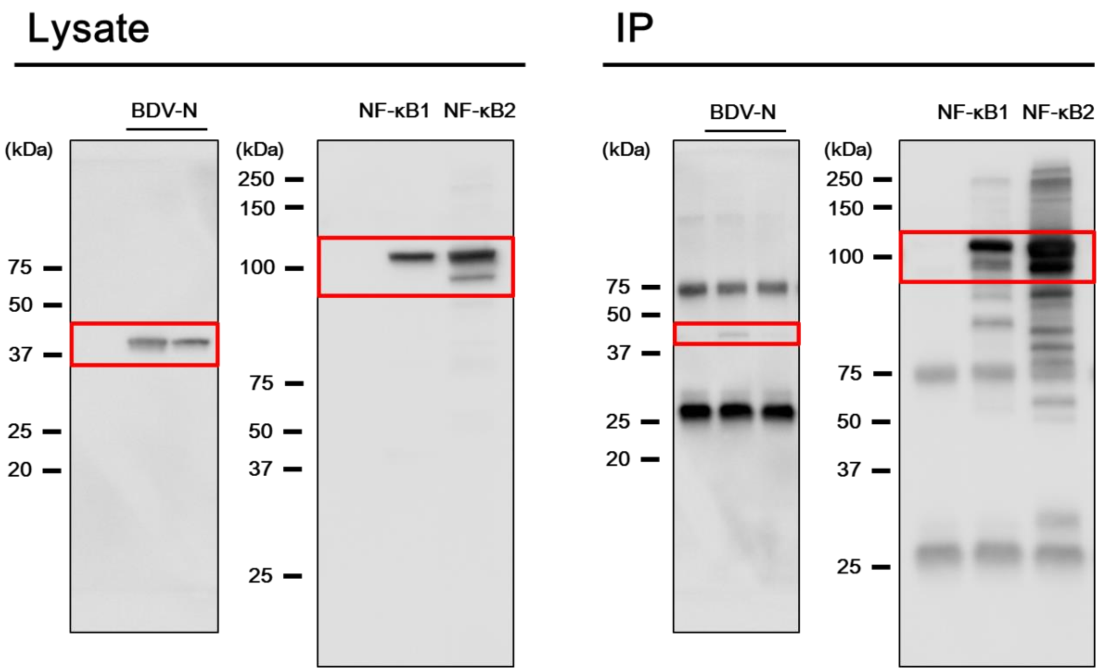

Figure S3 Full-length blots of Figure 4A

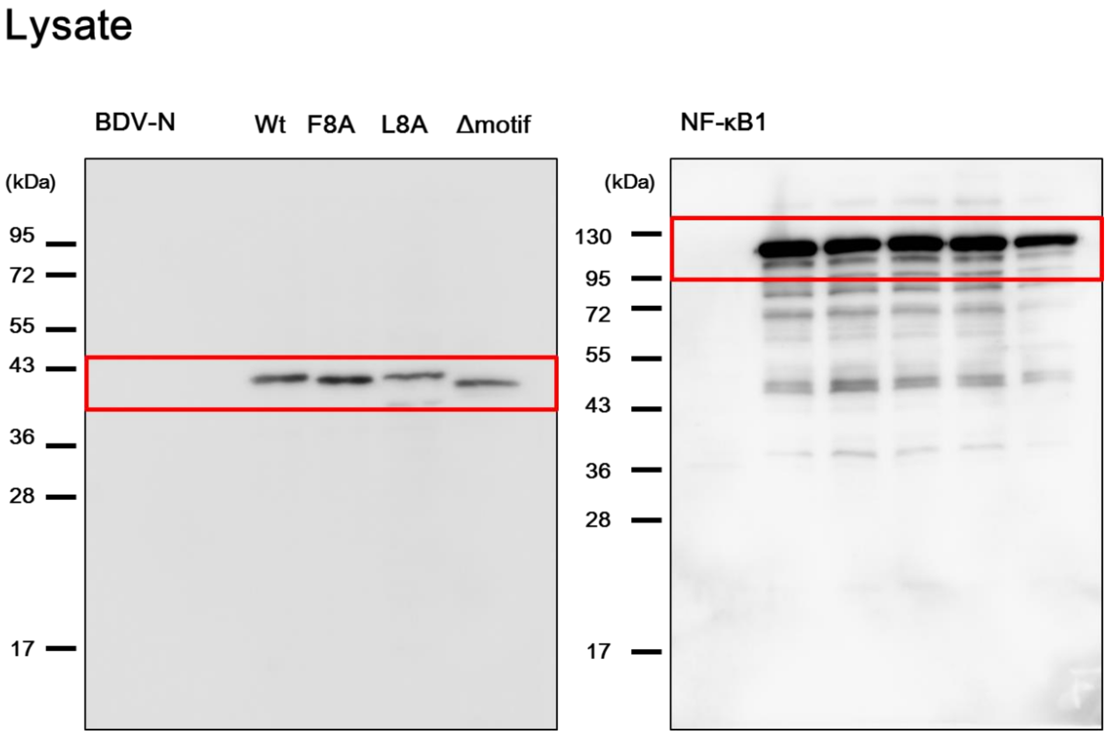

Figure S4A Full-length blots of cell lysates in Figure 4B

IP

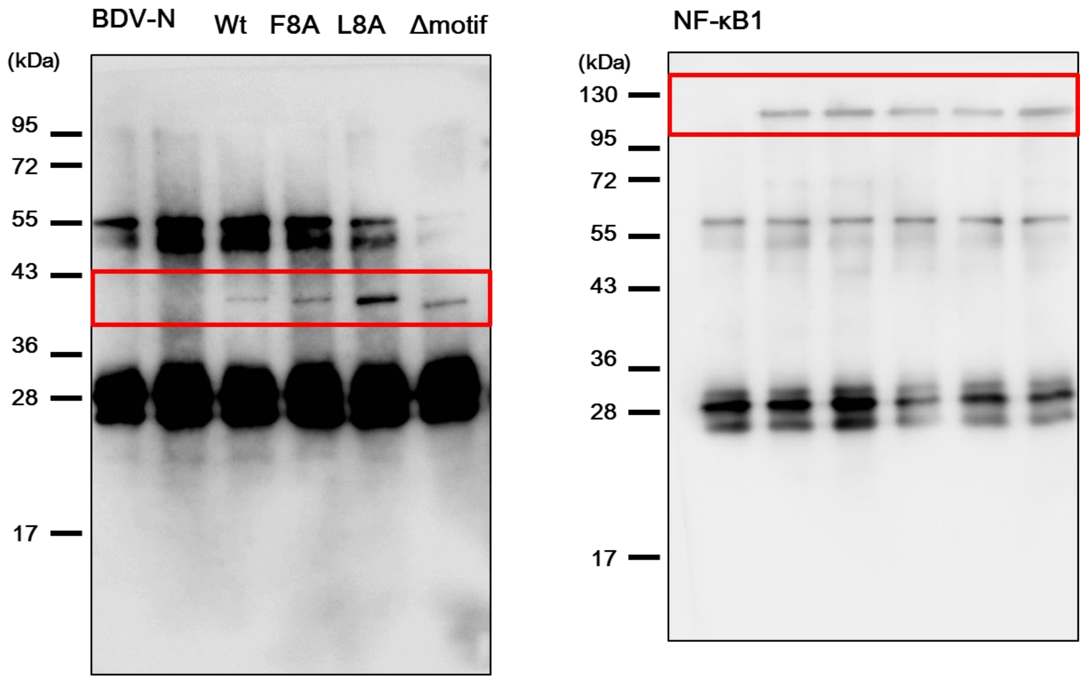

Figure S4B Full-length blots of IP products in Figure 4B
